# Development and validation of a prediction index for recent mortality in advanced COPD patients

**DOI:** 10.1038/s41533-021-00263-7

**Published:** 2022-01-13

**Authors:** Sheng-Han Tsai, Chia-Yin Shih, Chin-Wei Kuo, Xin-Min Liao, Peng-Chan Lin, Chian-Wei Chen, Tzuen-Ren Hsiue, Chiung-Zuei Chen

**Affiliations:** 1 Division of General Medicine, Department of Internal Medicine, National Cheng Kung University Hospital, College of Medicine, National Cheng Kung University, Tainan, Taiwan; 2 Department of Public Health, College of Medicine, National Cheng Kung University, Tainan, Taiwan; 3 Division of Pulmonary Medicine, Department of Internal Medicine, National Cheng Kung University Hospital, College of Medicine, National Cheng Kung University, Tainan, Taiwan; 4 Department of Internal Medicine and Oncology, National Cheng Kung University Hospital, College of Medicine, National Cheng Kung University, Tainan, Taiwan

**Keywords:** Outcomes research, Disease-free survival

## Abstract

The primary barrier to initiating palliative care for advanced COPD patients is the unpredictable course of the disease. We enroll 752 COPD patients into the study and validate the prediction tools for 1-year mortality using the current guidelines for palliative care. We also develop a composite prediction index for 1-year mortality and validate it in another cohort of 342 patients. Using the current prognostic models for recent mortality in palliative care, the best area under the curve (AUC) for predicting mortality is 0.68. Using the Modified Medical Research Council dyspnea score and oxygen saturation to define the combined dyspnea and oxygenation (DO) index, we find that the AUC of the DO index is 0.84 for predicting mortality in the validated cohort. Predictions of 1-year mortality based on the current palliative care guideline for COPD patients are poor. The DO index exhibits better predictive ability than other models in the study.

## Introduction

The prevalence of and mortality associated with chronic obstructive pulmonary disease (COPD) have been increasing annually^1^. However, current treatments have been disappointing in terms of controlling airflow obstructions and reducing mortality^2–4^. Although palliative care is shown to be effective in patients with COPD, these patients have fewer opportunities to receive palliative care than patients with cancer^5,6^. Jabbarian et al. found that the failure to implement advance care planning (ACP) in chronic diseases is mainly due to the complexity and unpredictability of the disease^7^, and the uncertainty of disease trajectory is even greater in COPD than in cancer^8–12^. In addition, COPD patients typically want to know more about their prognosis in the early stages^13,14^. Therefore, enormous effort has been made to find indicators to predict a poor prognosis accurately.

Researchers have found many indicators related to various adverse outcomes for COPD, including patient age, body mass index (BMI), dyspnea, smoking status, exercise capacity, acute exacerbation, symptoms, and biological indicators^15–17^. Unfortunately, as was the case with the first proposed indicator, FEV_1_, there was no optimal way to predict mortality based on the indicator^17,18^. After the multisystem involvement characteristic of COPD became known, the focus was moved to composite indicators to achieve better predictive outcomes^15,19^. The earliest developed and most widely investigated multicomponent indicators included the Body-Mass Index, Airflow Obstruction, Dyspnea, and Exercise Capacity (BODE) Index^20^, which was also recommended for predicting outcomes by the Global Initiative for Chronic Obstructive Lung Disease (GOLD)^21^. Later, numerous different indices were developed, including the Dyspnea and Airflow Obstruction (ADO) Index, the Dyspnea, Obstruction, Smoking, Exacerbation (DOSE) Index, and various modifications of the BODE index^22^. However, most of these indices were developed to predict long-term survival. They all lacked accuracy when applied to short-term events of <12 months^20–23^. Marin et al.^23^ validated a number of existing prognostic indices in a large individual pooled data set (*n* = 3633) from multiple cohort studies with different stages of COPD. These prognostic indices included the original BODE, the modified BODE (replacing the 6-min walk distance (6MWD) with peak oxygen uptake V’_O2_ as % predicted), the BODEx (replacing the 6MWD with exacerbations), the eBODE (BODE plus exacerbations), the SAFE (SGRQ score, air-flow limitation, and exercise tolerance), the ADO, and the DOSE. All-cause mortality prediction at 12 months was assessed for these indices, where the indices determined to be optimal for prediction was the ADO (*C* statistic = 0.70). Boeck et al.^24^ developed the B-AE-D indices (BMI, acute exacerbations, dyspnea) for 2-year mortality in the PROMISE study, and external validation of the B-AE-D was performed in COCOMICS and the COMIC study for 1-year all-cause mortality (*C* statistic = 0.68 and 0.74, respectively). Therefore, none of these indices had the strong predictive ability for 1-year mortality. In addition, none of these models were developed with the specific aim of predicting all-cause mortality in stable COPD patients within 12 months.

To the best of our knowledge, Bloom et al.^25^ was the only research group to develop indicators (the BARC index) for predicting 1-year mortality with the aim of palliative care in advanced COPD (*C* statistic = 0.78 and 0.70 for the development and validation cohorts, respectively). The variables in the BARC only required routinely collected non-specialist information, which, therefore, helped identify patients seen in primary care institutions, but a total of 18 variables were required. Because no existing indices had strong enough predictive ability for 1-year mortality in clinical practice, and very few indices were developed with the specific aim of predicting 1-year mortality for palliative care in stable COPD. In this study, we aimed to validate the currently recommended prediction indices for palliative care, we also developed a new predictive index for 1-year mortality in hospitalized ambulatory COPD patients.

## Methods

### Study design

We conducted this cohort study in the National Cheng Kung University Hospital (NCKUH) from August 2006 to December 2015. The patients included in the present study were part of another previous study^26^. The patients were eligible for inclusion if they had received regular management for COPD at our hospital for >1 year prior to their recruitment. All patients were diagnosed with COPD by pulmonologists according to the GOLD guidelines for diagnostic criteria^1^. The criteria were as follows: age >40 years, typical symptoms, such as cough, dyspnea, wheezing, or chest tightness in combination with evidence of chronic airflow obstruction, as defined by a postbronchodilator ratio of forced expiratory volume in 1 s (FEV_1_) to a forced vital capacity (FVC) of <70%. Pulmonary function tests were performed following the standard protocols of the American Thoracic Society^27^. All patients were enrolled under clinically stable conditions. We excluded patients who were unwilling to participate and those who had advanced lung cancer and pulmonary fibrosis because of anticipated death in the near future. Patients with missing data and those lost to follow-up in the first year were also excluded from the analysis. In total, 752 patients with COPD were analyzed (Supplementary Fig. 1). The Institutional Review Board of NCKUH approved this study before commencement (IRB number: B-ER-105-386 and B-ER-98-289). Written informed consent was obtained for all participants while enrollment.

### Prognostic variables and outcome

A total of 752 consecutive COPD patients were recruited. All patients were monitored through December 2016 or until death. We acquired age, smoking history, BMI, the severity of dyspnea assessed by grade on the modified Medical Research Council (mMRC) dyspnea scale^28^, the degree of comorbidity as evaluated using the Charlson index^29^, oxygen saturation levels as detected by pulse oximetry in room air (SpO_2_), and status of long-term home oxygen usage from every patient at the time of inclusion as determined by research assistants in the study. Comorbidity was evaluated using the Charlson index and included congestive heart failure, coronary artery disease, systemic hypertension, peptic ulcer, and diabetes mellitus as identified from the patient files and detailed interviews. A severe acute exacerbation of COPD was defined as an acute event characterized by a worsening of the patient’s respiratory symptoms that were beyond day-to-day variations that also required hospitalization. The number of severe exacerbations in the preceding year was recorded by research assistants according to the patient’s chart as the primary means of data collection; self-reported data was used to supplement this data.

All-cause mortality was defined as the endpoint of the study. The survival status of all patients was evaluated using a prospective observation, as reported in a previous study^26^. All patients were contacted during regular clinic visits or by telephone interviews (if they missed an appointment). Most patients who died during the study period had been regularly followed and had visited the hospital for treatment before their death. Their dates of death were recorded and verified using hospital records. Research assistants obtained the date of death of patients who died outside the hospital by telephone contact with partners or family members. Survival status was also verified through linkage with the Taiwan National Mortality Registry.

### Predictive variables for palliative care

In the first part of the study, we evaluated the predictive ability of the currently recommended variables for estimating 1-year mortality in the palliative care guideline for COPD. We selected several variables for building the predictive model based on a review of the currently recommended prediction variables^30–32^. The variables included (1) mMRC score = 4, (2) frequent, severe AE (two or more AEs requiring hospitalization in the preceding year), (3) hypoxemia (SpO_2_ < 90% in ambient air), (4) BMI < 21, and (5) predicted FEV_1_ < 30%. We used several combined indices to test the accuracy of the prediction for 1-year mortality. The patients were subdivided into four groups: Group 1 was defined as patients with frequent, severe AE in combination with severe dyspnea (mMRC = 4). Group 2 was defined as patients with frequent, severe AE in combination with SpO_2_ < 90% in ambient air. Group 3 was defined as patients with frequent, severe AE combined with predicted FEV_1_ < 30%. Group 4 was defined as patients with frequent, severe AE in combination with BMI < 21.

### Modeling the predictive scores

Because of the generally unsatisfactory predictive power found in previous studies and with validating our results, we wanted to derive a new predictive model for 1-year mortality from the patient variables, including age, sex, BMI, disease severity, such as mMRC dyspnea score, FEV_1_, SpO_2_, and comorbidities. The variables were evaluated using multivariate Cox regression models with a forward entering approach and a 5% significance level for the selection criteria. Significant regression coefficients were converted to exponential expressions for the weighting of the variables used for the predictive indices.

### Validation of the predicting index

To validate the predictive performance of our model, we selected a second cohort. All patients in the development group were recruited from pulmonary outpatient departments. Considering that if the validation group and the developmental group exhibited high homogeneity, it was expected that the proposed model would obtain very similar results for the two groups of patients. Patients in the validation group were recruited by screening individuals who had been diagnosed with COPD, not only in the pulmonary outpatient department but also in the Center for Hospice Palliative Shared Care at NCKUH from July 2012 to August 2019. All patients were aged ≥40 years; COPD was defined according to the GOLD diagnostic guidelines and criteria as the developmental group; patients with advanced lung cancer or pulmonary fibrosis were excluded. The date of recruitment of some patients from the Center for Hospice Palliative Shared Care overlapped with the time periods during which the development group was recruited. These patients were not excluded from this study since the source of patients was different from that for the development group (Center for Hospice Palliative Shared Care versus the pulmonary outpatient department). All patients had complete follow-up for 1 year or until death.

### Statistical analysis

Continuous variables are presented as the median and interquartile range because the number of deaths was not large and therefore may not follow a normal distribution. Therefore, comparisons between survivors and nonsurvivors were performed using Mann–Whitney *U*-test. Comparisons between categorical variables were performed using chi-square tests or Fisher’s exact tests.

Kaplan–Meier survival curves and log-rank tests were used for comparing different predictive variables. The ability to predict mortality within 1 year was analyzed using logistic regression models and the receiver operating characteristic (ROC) curve to calculate the area under the curve (AUC). Data processing and analyses were performed using the SPSS for Windows version 17.0 statistical software (IBM, Armonk, NY, USA).

### Reporting summary

Further information on research design is available in the Nature Research Reporting Summary linked to this article.

## Results

### Participants

We enrolled 752 COPD patients from August 2006 through December 2015. The mean age of the patients was 70.6 years, and most of them were men (92.7%). Twenty-eight percent of the patients had severe to very severe airflow limitations (FEV_1_ < 50% of predicted); 25.2% of the patients had mMRC scores from 3 to 4, and 50.7% had at least one severe AE in the preceding year during enrollment. At the end of the follow-up period in December 2016, 378 patients had died (50.3%), and 60 patients (8%) had died within 1 year after the start of follow-up.

The baseline characteristics of survivors and nonsurvivors are shown in Table 1. Compared with survivors, nonsurvivors were older, had a worse pulmonary function, lower BMI, lower oxygen saturation, worse symptoms of dyspnea (higher mMRC score), and more AEs in the previous year.

**Table 1:** Demographic and patient characteristics of survivors and nonsurvivors.

Characteristic^a^	Survivors (*n* = 692)	Nonsurvivors (*n* = 60)	*p* Value
Age, median (IQR)	71.2 (64.6, 78.7)	78.4 (72.5, 81.6)	<0.01
Male *n* (%)	640 (92.5)	57 (95.0)	0.61
Current smoker, *n* (%)	189 (27.3)	13 (21.7)	0.30
Smoking quantity (pack-years)	45 (23, 70)	50 (20, 62)	0.92
FEV_1_%	64 (48, 82)	50 (34, 64)	<0.01
BMI	23.3 (20.5, 25.8)	20.5 (17.0, 24.5)	<0.01
SpO_2_%	97.0 (95.0, 98.0)	95.5 (92.0, 97.0)	<0.01
CI score	2.0 (1.0, 3.0)	3.0 (1.0, 5.0)	<0.01
Severe AE ≥ 2, *n* (%)	111 (16.0)	22 (36.7)	<0.01
6MWT (meter)	344.0 (248.0, 400.0)	278.0 (206.0, 313.0)	0.11
SGRQ score	33.22 (18.2, 51.0)	59.13 (46.4, 65.3)	<0.01
mMRC = 4	26 (3.7)	17 (28.3)	<0.01
LTOT, *n* (%)	71 (10.3)	15 (25.0)	<0.01

^a^Discrete data are presented as number (percentage), and continuous variables are presented as median (IRQ).

*FEV*_*1*_ forced expiratory volume in 1 s, *BMI* body mass index, *SpO*_*2*_ oxygen saturation (%) detected with pulse oximeter when breathing in room air, *CI* Charlson index, *severe AE* *≥* *2 history* more than one acute exacerbation that required hospitalization in the preceding year, *6MWT* 6 min walking test, *SGRQ* St. George’s Respiratory Questionnaire, *mMRC* modified Medical Research Council Dyspnea Scale, *LTOT* long-term oxygen therapy.

### Predictive ability of the currently recommended models

**Fig. 1:**
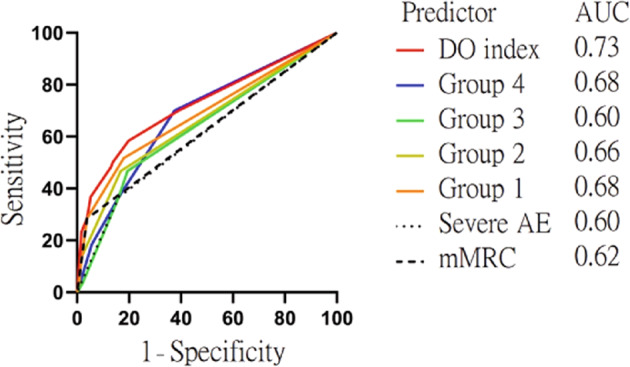
ROC curve for severe dyspnea, severe acute exacerbation, and different combinations of predictors and DO index for 1-year mortality in COPD patients. The AUC values for patients with severe dyspnea (mMRC = 4), frequent severe AE, groups 1–4, and DO index were 0.62, 0.60, 0.68, 0.66, 0.60, 0.68, and 0.73, respectively.

The AUC values for predicting 1-year mortality in patients with severe dyspnea (mMRC = 4) and patients having frequent, severe AE were 0.62 and 0.60, respectively. Combining predictor variables for patients with severe dyspnea and frequent severe AE was better than using only one variable (AUC = 0.68). The AUC for predicting 1-year mortality for patients with frequent, severe AE and SpO_2_ < 90% was 0.66. The AUC for patients with frequent, severe AE and predicted FEV_1_ < 30% was 0.60, and the AUC for patients with frequent, severe AE and BMI < 21 was 0.68. The ROC for the different composite indices did not differ significantly between the four groups of combinations (Fig. 1).

**Fig. 2:**
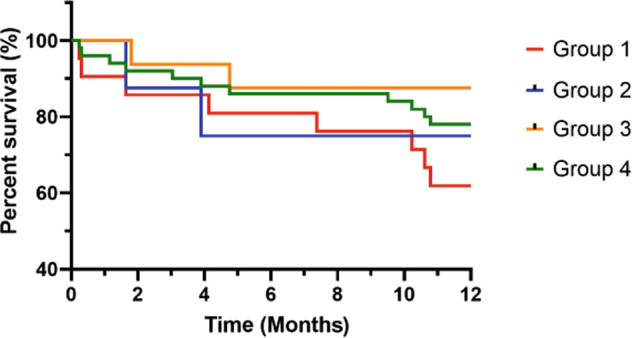
Kaplan–Meier survival curves for 1-year mortality according to different recommended prediction indices for palliative care. The 1-year survival rates were 62, 75, 88, and 78% for groups 1–4, respectively.

Kaplan–Meier survival analysis for all-cause mortality showed the 1-year survival rates for groups 1–4 were 62, 75, 88, and 78%, respectively (Fig. 2). All indices, including those for groups 1–4, showed high specificity but unsatisfactory sensitivity. The composite indices for group 1 with frequent, severe AE and severe dyspnea had a better positive predictive value than the other groups (Table 2).

**Table 2:** Predictive accuracy of different recommended palliative care indices for 1-year mortality.

Prognostic index	Sensitivity	Specificity	PPV	NPV	Accuracy	AUC
mMRC = 4	28.3%	96.2%	39.5%	93.9%	90.8%	0.623
Severe AE ≥ 2	36.7%	84.0%	16.5%	93.9%	80.2%	0.603
Group 1	13.3%	98.1%	38.1%	92.9%	91.4%	0.684
Group 2	3.3%	99.1%	25.0%	92.2%	91.5%	0.657
Group 3	3.3%	98.0%	12.5%	92.1%	90.4%	0.634
Group 4	18.3%	94.4%	22.0%	93.0%	88.9%	0.679

*mMRC* modified Medical Research Council Dyspnea Scale in stable condition, *Severe AE* *≥* *2* more than one acute exacerbation that required hospitalization in the preceding year, *Group 1* mMRC = 4 + severe AE ≥ 2, *Group 2* severe AE ≥ 2 + SpO_2_ < 90%, *Group 3* severe AE ≥ 2 + FEV_1_ < 30%, *Group 4* severe AE ≥ 2 + BMI < 21, *PPV* positive predictive value, *NPV* negative predictive value, *AUC* area under the curve, *SpO*_*2*_ oxygen saturation (%) detected with a pulse oximeter when breathing room air, *FEV*_*1*_ forced expiratory volume in 1 s, *BMI* body mass index.

### Development and validation of a prediction index

In the univariate analysis, there were significant differences between survivors and nonsurvivors in age, FEV_1_, SpO_2_, mMRC, BMI, anemia, and concurrent malignancies. Using multivariate regression, only mMRC and SpO_2_ were independent risk factors for predicting 1-year mortality.

We refined the dyspnea and oxygenation (DO) index by weighting dyspnea and SpO_2_ based on the results of the multivariate regression model. We used the integers closest to the hazard ratio for scoring predictive variables (Table 3). Compared with groups 1–4, the DO index had better discrimination for mortality (AUC = 0.73; Fig. 1). We also performed a sensitivity analysis for patients with severe or very severe obstruction (FEV_1_ < 50%), where the DO index performed better (AUC = 0.81). The survival rates for the different DO scores are shown in Table 4. In the group with the most severe COPD (DO score = 12–16), the survival rate was only 20%.

**Table 3:** Weighting of variables in DO index.

Variable	*β*	Adjusted HR	Score
SpO_2_ (%)			
95–100	0	1	1
90–94	0.55	1.7	2
85–89	1.05	2.9	3
<85	2.00	7.3	7
mMRC score			
0–2	0	1	1
3	0.64	1.9	2
4	2.21	9.1	9

Coding according to the regression coefficient for DO index construction.

*DO* dyspnea and oxygenation.

**Table 4:** Survival analysis of 1-year mortality for different DO index scores of patients with severe and very severe COPD (*n* = 180).

Score	Survived (*n*)	Died (*n*)	Survival rate (%)	Statistics (chi-square)	*p* Value^a^
DO				58.61	<0.001
2	56	2	96.55		
3	58	2	96.67		
4	30	3	90.91		
5	2	0	100.00		
8	1	0	100.00		
9	0	1	0		
10	10	2	83.3		
11	4	4	50		
12	1	2	33.3		
16	0	2	0		

Score	Survived (*n*)	Died (*n*)	Survival rate (%)	Statistics (chi-square)	*p* Value^b^
DO				52.98	<0.001
2–7	147	7	95.45		
8–11	14	7	66.7		
12–16	1	4	20.00		

^a^Wilcoxon test.

^b^Log-rank test.

We enrolled a total of 342 patients for the validation group. The patients in the validation group were older (73.5 vs. 72.2%), had lower oxygenation (96 vs. 97%), more comorbidities, as evaluated by the CI score (4.0 vs. 2.0%), more symptoms, as evaluated by the percentage of patients with mMRC = 4 (14.3 vs. 6.3%), and higher 1-year mortality (14.3 vs. 8.0%), than the developmental group (Supplementary Table 1). When applying our DO score for predicting 1-year mortality, the AUC was 0.84. In the group with the most severe COPD (DO score = 12–16), the positive and negative predictive values were 87% and 89%, respectively, for 1-year mortality.

## Discussion

This study showed that a combination of mMRC and frequent, severe AE as a predictor of 1-year mortality demonstrated similar poor discrimination power as other combinations of factors. These factors included predictors with desaturation, or a poor grade of lung function, or low BMI combined with frequent, severe AE. The AUC values of these combinations ranged from 0.60 to 0.68. In addition, all combined groups exhibited lower sensitivity but higher specificity. Therefore, these indices did a good job of ruling outpatients who would survive for >1 year but tended to miss patients who would die within 1 year.

Using the mMRC dyspnea score and oxygen saturation (SpO_2_), we developed a better discriminating model for 1-year mortality, the DO index. The AUC value of the DO index was 0.73 for the prediction of 1-year mortality, and the AUC was 0.84 in the validated cohort, which was superior to the current palliative guideline-recommended prediction tool. There was no process for sample size calculation in the study. Therefore, we calculated the statistical power backward using our sample size. According to the predictive ability of existing indices in a previous review^23–25^, we considered an AUC of 0.65 as the median discrimination power for previous predictors. Using our population of a total of 342 patients in the validation group for a two-sided *z*-test at a significance level of 0.05, we achieved a 99% power to detect the AUC between the median discrimination power of previous predictors and the DO index in this study (PASS Power Analysis and Sample Size Software, NCSS, LLC., Kaysville, Utah). However, there were only 18 deaths out of 180 patients when the DO index was applied in severe COPD patients (see Table 4). The small numbers in some lattices may have thus affected the accuracy of the estimate. For example, when using a DO score = 9 as a cutoff value, the 1-year survival rate for patients with DO scores ≥ 9 was estimated to be 58% (15/26). The accuracy of the predictive ability, sensitivity, specificity, positive predictive value, and negative predictive value was 87.8% (95% CI (confidence interval), 82.1–92.2%), 61.1% (95% CI, 35.8–82.7), 90.7% (95% CI, 85.2–94.7), 42.3% (95% CI, 28.6–57.4), and 95.5% (95% CI, 92.2–97.4), respectively. The low precision for sensitivity and the positive predictive value as indicated by wider confidence intervals was attributed to the smaller sample size in this test.

In general, the discriminative power of the model for the development group was better than that for the validation group. Patients in the validation group were older, had lower oxygenation, more comorbidities, more symptoms, and higher mortality than the development group in this study. The heterogeneity between our developmental and validation groups was one of the reasons explaining why the predictive ability was better in the validation group than in the development group. To evaluate whether the predictive ability of the DO index becomes stronger over time, we analyzed the predictive ability for 3- and 5-year mortality and found that the AUC values were 0.66 and 0.67, respectively. These results implied that the DO index is not suitable for predicting 3- and 5-year mortality.

The findings of poor discrimination for the current models in this study were consistent with a previous systemic review conducted by Almagro et al.^33^. They used indicators already developed in previous articles to validate the performance in their cohort. Composite indices were better than a single parameter, and the best AUC was 0.68 for the CODEX index (comorbidity, obstruction, dyspnea, and previous exacerbation). The author concluded that no single index is good enough to guide the initiation of palliative care. Thus, the clinician should not make this decision based solely on a predictive tool. However, the use of the proposed DO index improved the predictive power (AUC = 0.73) for 1-year mortality, and the AUC was 0.84 in the validated cohort. The BARC index for prognostic factors, including BMI and blood results (B), age (A), respiratory variables (airflow obstruction, exacerbations, smoking) (R), and comorbidities (C) was conducted based on medical databases and had a satisfactory AUC for 1-year mortality (AUC = 0.79). However, it included 18 variables in the model, such as age, BMI, FEV_1_, severe exacerbations, smoking status, multiple comorbidities, hemoglobin, platelets, and others for the evaluation^34^. In contrast, the DO index proposed in this study is simple to use because only two clinical parameters, dyspnea score and oxygenation, detected with a pulse oximeter, are needed. Another composite index, the ProPal-COPD tool, had a good predictive ability for 1-year mortality with an AUC of 0.82. This model relied on the following seven predictors: (1) a surprise question, (2) MRC dyspnea, (3) the Clinical COPD Questionnaire (CCQ), (4) FEV_1_% of predicted value, (5) BMI, (6) previous hospitalizations for AECOPD, and (7) specific comorbidities. However, some variables, such as the surprise question and CCQ, were not always routinely captured.

The DO index developed in this study was composed of the mMRC score and oxygen saturation. In a recent systematic review of predictive indicators in COPD, 24 models used composite indicators^35^. The dyspnea score (mMRC) is one of the ten most used parameters. Nishimura et al. also demonstrated that the severity of dyspnea was a more favorable predictor of death than FEV_1_^17^. This result was consistent with the use of mMRC in this article. Our model also included another indicator, SpO_2_, which was not commonly used in previous studies. Instead, some studies used arterial oxygen partial pressure (PaO_2_) as a predictor. PaO_2_ is most often used during hospitalization, where PaO_2_ can fluctuate due to many factors, such as oxygen use, pneumonia, or cardiovascular instability. Additionally, drawing arterial blood may also lead to some local complications. In our study, we used SpO_2_ in stable patients breathing ambient air to measure constant oxygenation status. Lower levels of invasiveness are also preferred in outpatient settings.

The strength of this study is its diagnostic and measurement accuracy. COPD was diagnosed according to standard evaluations and spirometry results, and the deaths were verified by linking with a database from the Taiwan National Mortality Registry. In addition, we performed a sensitivity analysis by excluding a small amount of missing mMRC values and by restricting the subjects to patients with severe airflow limitations. Both results showed similar findings, which indicated the consistency of the discriminative validity. Finally, we validated the DO index and showed its good predictive ability for 1-year mortality in the second cohort of 342 patients.

There are limitations to our study. First, we did not have a large patient population and were only limited to one medical center. The second limitation of the present study was that few women (7.3%) were enrolled. Recently, a large, real-world, cohort study revealed gender differences among COPD patients, where COPD was more frequent among women (53.8%), and the overall mortality rate was higher in men as compared with women (45 vs. 38%). However, no differences in mortality due to COPD related to gender were found^36^. The majority of participants with smoking-related COPD in Taiwan are male^37^; the smoking prevalence for women is <5% in Taiwan. In contrast to industrialized countries in the West, COPD morbidity remains male predominant in most Asian countries^37–39^. However, the result of this study cannot be directly generalizable to other countries due to this limitation.

The third limitation was that socioeconomic status (SES) was not included in our predictive model. SES disadvantages appear to have a significant impact on COPD mortality and morbidity, where individuals with the lowest SES consistently had been shown to have significantly higher mortality than those with the highest SES^40,41^. We thus suggest including an SES measurement in the predictive model in further studies.

In conclusion, this study demonstrated that the predictive values for 1-year mortality were poor, based on the current recommendations for palliative care among COPD patients, including four different composite indices. The newly developed DO index proposed in this work exhibited better predictive ability than other alternatives. We suggest that COPD patients with DO index scores ≥12, for example, patients with mMRC = 4 and SpO_2_ < 90%, are good candidates to receive palliative care.

## Supplementary information

REPORTING SUMMARY

Supplementary table 1 and figure 1

## Data Availability

Full data sets are not available publicly currently for protecting patient privacy. But the data can be requested reasonably to the corresponding author.
