# Supplementary material for: Development and validation of a prediction index for recent mortality in advanced COPD patients
Source: NPJ Prim Care Respir Med. 2022 Jan 13;32:2. doi: 10.1038/s41533-021-00263-7 (PMC8758667; doi:10.1038/s41533-021-00263-7)
Supplement: Supplementary file 1 — REPORTING SUMMARY [file 41533_2021_263_MOESM1_ESM.pdf]

nature portfolio

Corresponding author(s): Chiung-Zuei Chen

Last updated by author(s): Aug 23, 2021

Reporting Summary

Nature Portfolio wishes to improve the reproducibility of the work that we publish. This form provides structure for consistency and transparency in reporting. For further information on Nature Portfolio policies, see our Editorial Policies and the Editorial Policy Checklist.

Statistics

For all statistical analyses, confirm that the following items are present in the figure legend, table legend, main text, or Methods section.

n/a	Confirmed	
☐	☒	The exact sample size (*n*) for each experimental group/condition, given as a discrete number and unit of measurement
☐	☒	A statement on whether measurements were taken from distinct samples or whether the same sample was measured repeatedly
☐	☒	The statistical test(s) used AND whether they are one- or two-sided<br>*Only common tests should be described solely by name; describe more complex techniques in the Methods section.*
☐	☒	A description of all covariates tested
☒	☐	A description of any assumptions or corrections, such as tests of normality and adjustment for multiple comparisons
☐	☒	A full description of the statistical parameters including central tendency (e.g. means) or other basic estimates (e.g. regression coefficient) AND variation (e.g. standard deviation) or associated estimates of uncertainty (e.g. confidence intervals)
☐	☒	For null hypothesis testing, the test statistic (e.g. *F*, *t*, *r*) with confidence intervals, effect sizes, degrees of freedom and *P* value noted<br>*Give P values as exact values whenever suitable.*
☒	☐	For Bayesian analysis, information on the choice of priors and Markov chain Monte Carlo settings
☒	☐	For hierarchical and complex designs, identification of the appropriate level for tests and full reporting of outcomes
☒	☐	Estimates of effect sizes (e.g. Cohen's *d*, Pearson's *r*), indicating how they were calculated

Our web collection on statistics for biologists contains articles on many of the points above.

Software and code

Policy information about availability of computer code

Data collection	*Provide a description of all commercial, open source and custom code used to collect the data in this study, specifying the version used OR state that no software was used.*
Data analysis	*Provide a description of all commercial, open source and custom code used to analyse the data in this study, specifying the version used OR state that no software was used.*

For manuscripts utilizing custom algorithms or software that are central to the research but not yet described in published literature, software must be made available to editors and reviewers. We strongly encourage code deposition in a community repository (e.g. GitHub). See the Nature Portfolio guidelines for submitting code & software for further information.

Data

Policy information about availability of data

All manuscripts must include a data availability statement. This statement should provide the following information, where applicable:

- Accession codes, unique identifiers, or web links for publicly available datasets
- A description of any restrictions on data availability
- For clinical datasets or third party data, please ensure that the statement adheres to our policy

The datasets generated during and/or analysed during the current study are available from the corresponding author on reasonable request.

Field-specific reporting

Please select the one below that is the best fit for your research. If you are not sure, read the appropriate sections before making your selection.

☒ Life sciences ☐ Behavioural & social sciences ☐ Ecological, evolutionary & environmental sciences

For a reference copy of the document with all sections, see nature.com/documents/nr-reporting-summary-flat.pdf

Life sciences study design

All studies must disclose on these points even when the disclosure is negative.

Sample size	There was no process for sample size calculation in the study. Therefore, we calculated the statistical power backward using our sample size. According to the predictive ability of existing indices in a previous review , we considered an AUC of 0.65 as the median discrimination power for previous predictors. Using our population of a total 342 patients in the validation group for a two-sided z-test at a significance level of 0.05, we achieved a 99% power to detect the AUC between the median discrimination power of previous predictors and the DO index in this study (PASS Power Analysis and Sample Size Software, NCSS, LLC. Kaysville, Utah).
Data exclusions	We excluded patients who were unwilling to participate and those who had advanced lung cancer and pulmonary fibrosis because of anticipated death in the near future. Patients with missing data and those lost to follow up in the first year were also excluded from the analysis
Replication	To validate the predictive performance of our model, we selected a second cohort, the AUC was 0.84 in the validated cohort.
Randomization	Non randomized clinical trial
Blinding	Non randomized clinical trial

Reporting for specific materials, systems and methods

We require information from authors about some types of materials, experimental systems and methods used in many studies. Here, indicate whether each material, system or method listed is relevant to your study. If you are not sure if a list item applies to your research, read the appropriate section before selecting a response.

Materials & experimental systems

n/a	Involved in the study	
☒	☐	Antibodies
☒	☐	Eukaryotic cell lines
☒	☐	Palaeontology and archaeology
☒	☐	Animals and other organisms
☐	☒	Human research participants
☒	☐	Clinical data
☒	☐	Dual use research of concern

Methods

n/a	Involved in the study	
☒	☐	ChIP-seq
☒	☐	Flow cytometry
☒	☐	MRI-based neuroimaging

Human research participants

Policy information about studies involving human research participants

Population characteristics	We enrolled 752 COPD patients from August 2006 through December 2015. The mean age of the patients was 70.6 years, and most of them were men (92.7%). Twenty-eight percent of the patients had severe to very severe airflow limitations (FEV1 < 50% of predicted); 25.2% of the patients had mMRC scores from 3-4, and 50.7% had at least one severe AE in the preceding year during enrollment. At the end of the follow-up period in December 2016, 378 patients had died (50.3%), and 60 patients (8%) had died within 1 year after the start of follow-up.
Recruitment	We conducted this cohort study in National Cheng Kung University Hospital from August 2006 to December 2015. The patients included in the present study were part of another previous study [26]. The patients were eligible for inclusion if they had received regular management for COPD at our hospital for more than 1 year prior to their recruitment. All patients were diagnosed with COPD by pulmonologists according to the GOLD guidelines for diagnostic criteria.
Ethics oversight	The Institutional Review Board of National Cheng Kung University Hospital (NCKUH) approved this study before commencement (IRB number: B-ER-105-386 and B-ER-98-289).

Note that full information on the approval of the study protocol must also be provided in the manuscript.
