# Supplementary material for: Development and validation of a prediction index for recent mortality in advanced COPD patients
Source: NPJ Prim Care Respir Med. 2022 Jan 13;32:2. doi: 10.1038/s41533-021-00263-7 (PMC8758667; doi:10.1038/s41533-021-00263-7)
Supplement: Supplementary file 2 — Supplementary table 1 and figure 1 [file 41533_2021_263_MOESM2_ESM.pdf]

Supplementary table 1
Mortality and Patients' characteristics of developmental and validation groups

**Characteristic***	**Developmental group (n = 750)**	**Validation group (n = 342)**	**p-Value**
Mortality	60 (8.0)	49 (14.3)	< 0.01
Age, median (IQR)	72.2 (65, 78.9)	73.5 (66.6, 81.1)	< 0.01
Male, n (%)	697 (92.9)	316 (92.4)	0.87
Current smoker, n (%)	202 (26.9)	65 (19.0)	< 0.01
FEV_1%	63 (48, 81)	66 (49, 81)	0.52
BMI	23.1 (20.2, 25.8)	23.4 (20.3, 26.6)	0.29
SpO_2%	97 (95.0, 98.0)	96 (94.0, 98.0)	< 0.01
CI score	2.0 (1.0, 3.0)	4.0 (4.0, 6.0)	< 0.01
Severe AE ≥ 2, n (%)	133 (17.7)	42 (12.3)	0.02
mMRC = 4, n (%)	43 (5.7)	49 (14.3)	< 0.01

*Discrete data are presented as number (percentage), and continuous variables are presented as median (IRQ).

Abbreviations: FEV_1, forced expiratory volume in 1 second; BMI, body mass index; SpO_2, oxygen saturation (%) detected with pulse oximeter when breathing in room air; CI, Charlson index; severe AE ≥ 2 history, more than one acute exacerbation required hospitalization in the preceding year; 6MWT, 6 minutes walking test; SGRQ, St. George's Respiratory Questionnaire; mMRC, modified Medical Research Council Dyspnea Scale

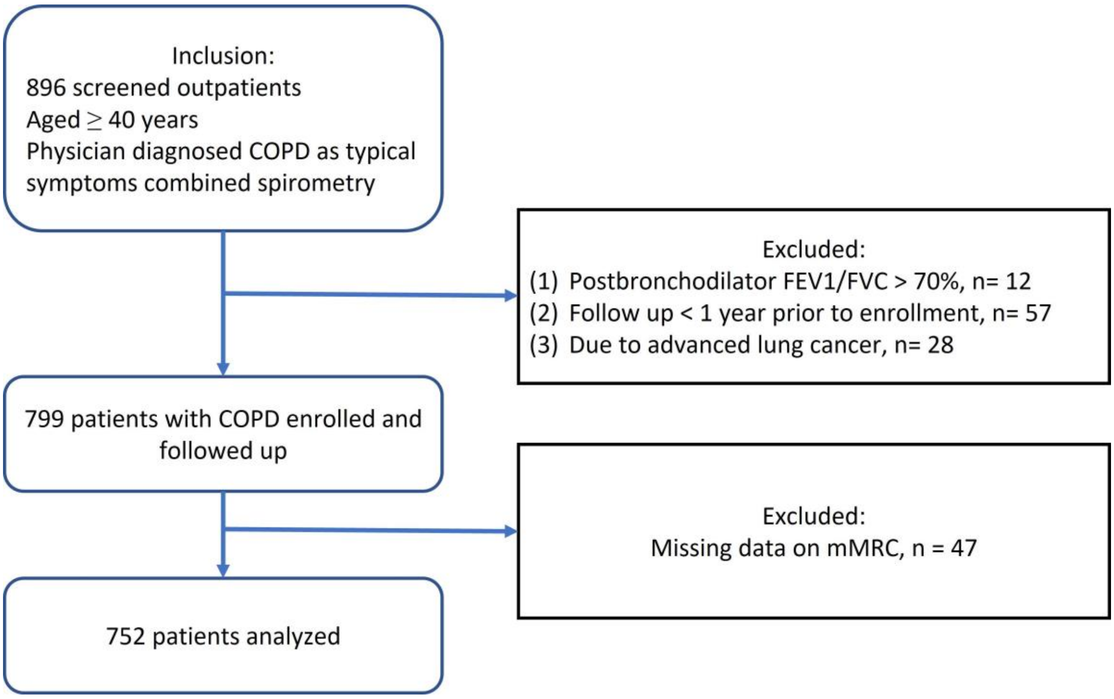

Supplementary figure 1. Flowchart of participants' recruitment
